# Parental origin of the allotetraploid tobacco *Nicotiana benthamiana*

**DOI:** 10.1111/tpj.14648

**Published:** 2020-01-13

**Authors:** Matteo Schiavinato, Marina Marcet‐Houben, Juliane C. Dohm, Toni Gabaldón, Heinz Himmelbauer

**Affiliations:** ^1^ Department of Biotechnology Institute of Computational Biology University of Natural Resources and Life Sciences (BOKU) 1190 Vienna Austria; ^2^ Bioinformatics and Genomics Programme Centre for Genomic Regulation (CRG) The Barcelona Institute of Science and Technology 08003 Barcelona Spain; ^3^ ICREA Barcelona Spain; ^4^ Present address: Barcelona Supercomputing Centre (BSC‐CNS) and Institute for Research in Biomedicine (IRB) Barcelona Spain

**Keywords:** *Nicotiana benthamiana*, tobacco, interspecific hybrid, subgenome separation, phylome analysis, plant genomics, phylogenetic tree

## Abstract

*Nicotiana* section *Suaveolentes* is an almost all‐Australian clade of allopolyploid tobacco species including the important plant model *Nicotiana benthamiana.* The homology relationships of this clade and its formation are not completely understood. To address this gap, we assessed phylogenies of all individual genes of *N. benthamiana* and the well studied *N. tabacum* (section *Nicotiana*) and their homologues in six diploid *Nicotiana* species. We generated sets of 44 424 and 65 457 phylogenetic trees of *N. benthamiana* and *N. tabacum* genes, respectively, each collectively called a phylome. Members of *Nicotiana* sections *Noctiflorae* and *Sylvestres* were represented as the species closest to *N. benthamiana* in most of the gene trees. Analyzing the gene trees of the phylome we: (i) dated the hybridization event giving rise to *N. benthamiana* to 4–5 MyA, and (ii) separated the subgenomes. We assigned 1.42 Gbp of the genome sequence to section *Noctiflorae* and 0.97 Gbp to section *Sylvestres* based on phylome analysis. In contrast, read mapping of the donor species did not succeed in separating the subgenomes of *N. benthamiana*. We show that the maternal progenitor of *N. benthamiana* was a member of section *Noctiflorae*, and confirm a member of section *Sylvestres* as paternal subgenome donor. We also demonstrate that the advanced stage of long‐term genome diploidization in *N. benthamiana* is reflected in its subgenome organization. Taken together, our results underscore the usefulness of phylome analysis for subgenome characterization in hybrid species.

## Significance Statement

*Nicotiana benthamiana* is a tetraploid plant that arose by interspecific hybridization. We constructed the *N. benthamiana* phylome, that is a large collection of gene trees, analyzed the species' ancestry, dated the hybridization event, and assigned most of the genome to its parental origin. By phylome analysis, subgenome separation succeeded when classical mapping‐based methods failed due to an advanced stage of re‐establishing a diploid state in the *N. benthamiana* genome.

## Introduction

**Figure 1:**
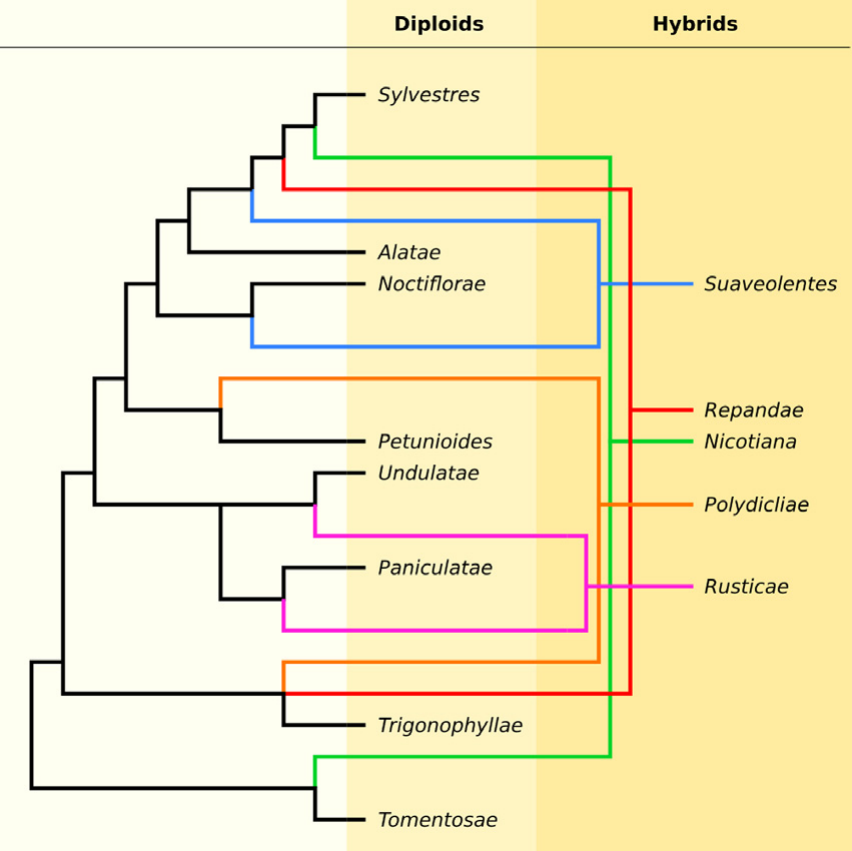
Representation of the *Nicotiana* sections and hybrids. Tree branches are intended to show relationships but are not scaled to actual phylogenetic distance. Outline and tree topology are based on a previous study (Knapp *et al.*, 2004). Black branches indicate the evolution of diploid taxa, coloured branches refer to the evolution of hybrid taxa.

*Nicotiana benthamiana* is an Australian tobacco species, mostly popular as a platform for recombinant protein production (van Herpen *et al.*, 2010; Bally *et al.*, 2018). The *Nicotiana* genus is part of the *Solanaceae*, a family that includes many economically relevant plants such as tomato, potato, and eggplant. The genus *Nicotiana* is organized into several sections, five of which contain polyploids formed by interspecific hybridization (Figure 1; Knapp *et al.*, 2004; Leitch *et al.*, 2008). Among these, *N. benthamiana* belongs to the section *Suaveolentes*, an almost all‐Australian clade with the exception of a few species that are native to Africa, New Caledonia or French Polynesia.

It has repeatedly been shown that the paternal progenitor in the hybridization that resulted in the species of section *Suaveolentes* is affiliated with the section *Sylvestres *(Knapp *et al.*, 2004; Leitch *et al.*, 2008; Clarkson *et al.*, 2010; Wang and Bennetzen, 2015; Bally *et al.*, 2018). The origin of the maternal progenitor is less clear, and different suggestions have been made depending on which phylogenetic markers were used (Leitch *et al.*, 2008; Kelly *et al.*, 2012; Clarkson *et al.*, 2017). A certain preference for section *Noctiflorae* has been consistent throughout the literature, mostly based on analyses involving multiple regions of the *N. benthamiana* plastid genome which is maternally inherited (Aoki and Ito, 2000; Clarkson *et al.*, 2004). However, none of the previous studies could yet clearly resolve the maternal phylogeny.

The date of the hybridization event has been estimated multiple times. It was believed that section *Suaveolentes* originated more than 10 million years ago (MyA; Clarkson *et al.*, 2004; Leitch *et al.*, 2008; Kelly *et al.*, 2012; Bally *et al.*, 2018). However, another study dated the hybridization event at 6 MyA, followed by a lag phase whereby the original hybrid expanded throughout the Australian territory, favoured by a humid climate. During the Pleistocene, the aridification of Australia coincided with a radiation of the clade that has led to more than 70 extant *Suaveolentes* species (Clarkson *et al.*, 2017). The positive response towards aridification is a rare and qualifying property, attributed to the uncommon genome plasticity of species from this section (Bally *et al.*, 2015). *Nicotiana* hybrid species show a high level of intermixing between their subgenomes (Lim *et al.*, 2007). Tobacco species affiliated with section *Suaveolentes* are likely to be the oldest *Nicotiana* polyploids (Clarkson *et al.*, 2004) in an advanced stage of long‐term genome diploidization. Many members of this section have fewer than *n* = 24 chromosomes, which is less than the sum of the chromosomes of their parents (*n* = 12; Leitch *et al.*, 2008). A high genetic turnover has been shown in some *Nicotiana* hybrids (Lim *et al.*, 2007), involving intense mobilization of retrotransposons (Petit *et al.*, 2010) and genome downsizing, mostly affecting the paternally derived subgenome (Renny‐Byfield *et al.*, 2011). *N. benthamiana* is therefore expected to have undergone extensive rearrangements among its two subgenomes.

Our study aimed to address the open questions on the maternal progenitor of *N. benthamiana* and the time of the interspecific hybridization event that gave rise to *N. benthamiana*. We address both questions using a high‐throughput phylogenetic approach involving the reconstruction of phylogenetic trees for all genes encoded in a genome, referred to as a phylome (Huerta‐Cepas *et al.*, 2007). By inspecting the topologies of individual gene trees, we infer the most likely pair of parents for *N. benthamiana* and use this information to separate its subgenomes. We validated our strategy by comparisons with *Nicotiana tabacum*, for which the details of the hybridization are known (Sierro *et al.*, 2014; Edwards *et al.*, 2017).

## Results

### Selection and preparation of Nicotiana gene sets

All the polyploid species in the *Nicotiana* genus originated from diploid *Nicotiana* parents (Clarkson *et al.*, 2004; Kelly *et al.*, 2012). Hence, we studied the parental origin of *N. benthamiana* through its homology relationships with diploid *Nicotiana* species each representing a different section. We included four publicly available gene sets from diploid tobacco species in our analysis, that is from *N. attenuata* and *N. obtusifolia (*Xu *et al.*, 2017), as well as from *N. sylvestris* and *N. tomentosiformis* (Sierro *et al.*, 2013)*.* Gene sets from representatives of two additional sections, *N. cordifolia* and *N. noctiflora*, were assembled by us using previously generated mRNA‐Seq data from leaf tissue (Long *et al.*, 2016). After quality‐trimming we assembled 40 million read‐pairs for *N. cordifolia* and 89 million read‐pairs for *N. noctiflora* into an initial set of 147 902 *N. cordifolia* transcripts (avg. length 1127 nt) and 168 632 *N. noctiflora* transcripts (avg. length 1026 nt)*.* The final gene sets contained 22 251 genes for *N. cordifolia* (avg. length 1480 nt) and 22 940 genes for *N. noctiflora* (avg. length 1494 nt; Table 1). We assessed the completeness by homology searches to 1440 highly conserved plant genes using BUSCO (Simão *et al.*, 2015) of which 75% were matched in the assembled transcriptome of *N. cordifolia* and 78% were matched in the assembled *N. noctiflora* transcriptome. These values, which are below a fully satisfactory BUSCO score, can be explained by the absence of tissue diversity as the *N. cordifolia* and *N. noctiflora* transcript sequences were exclusively derived from leaf samples. Nevertheless, we considered that these *de novo* assembled gene sets would provide a reasonably good representation of the transcriptomes of *N. cordifolia* and *N. noctiflora*.

**Table 1:** Species and data representing the eight sections of the genus *Nicotiana* analyzed in this study. Column ‘Analyzed’ indicates the number of genes uploaded into PhylomeDB for phylome construction. Identical sequences were merged (column ‘Removed’). SRA codes correspond to the raw mRNA‐Seq data used for the transcriptomic coverage analysis and for gene set calculation (in case of *N. cordifolia*, *N. noctiflora*)

				Transcripts			mRNA‐Seq data
Species	NCBI Taxid	Section	Ploidy level	Raw	Analyzed	Removed	SRA code
*N. attenuata*	49451	*Petunioides*	2	33 449	32 968	481	SRR1950890
*N. benthamiana*	4100	*Suaveolentes*	4	50 503	50 090	413	SRR7540371
*N. cordifolia*	140890	*Paniculatae*	2	22 251	22 242	9	SRR2106516
*N. noctiflora*	118707	*Noctiflorae*	2	22 940	22 928	12	SRR2106514
*N. obtusifolia*	200316	*Trigonophyllae*	2	28 147	27 794	353	SRR2912995
*N. sylvestris*	4096	*Sylvestres*	2	39 450	32 877	6573	ERR274390
*N. tabacum*	4097	*Nicotiana*	4	69 500	69 323	177	–
*N. tomentosiformis*	4098	*Tomentosae*	2	35 770	31 121	4649	SRR2106531

Additionally, we included the gene sets from *N. benthamiana *(Schiavinato *et al.*, 2019) and from *N. tabacum* (Edwards *et al.*, 2017) comprising 50 503 and 69 500 protein‐coding genes, respectively. *N. tabacum* was used as control throughout our study as it is a well‐characterized hybrid with known parents (namely *N. sylvestris* and *N. tomentosiformis*); the hybridization event has been dated between 0.2 and 0.4 MyA (Sierro *et al.*, 2014; Edwards *et al.*, 2017). Our final collection featured six diploid *Nicotiana* species (*N. attenuata, N. cordifolia, N. noctiflora, N. obtusifolia, N. sylvestris* and *N. tomentosiformis*) and two allotetraploid species (*N. benthamiana* and *N. tabacum*; Table 1).

### Phylome reconstruction

Using the PhylomeDB pipeline (Huerta‐Cepas *et al.*, 2014) we built a phylome for *N. benthamiana* and one for *N. tabacum* using their gene sets as seeds to study homology relationships between their genes and those of the six diploid *Nicotiana* species (Table 1)*.* PhylomeDB requires at least three homologues within the data set (orthologues or paralogues), to build a gene tree, a condition that was true for 44 424 (88.7%) *N. benthamiana* seed genes, representing the backbone phylome. The remaining 5666 genes did not return a tree for various reasons: 281 genes had no homologues, 3390 had only one or two homologues, and 1995 genes showed premature stop codons within the coding sequence (CDS). The latter can be attributed to result from technical issues, such as problems of sequencing data quality, or artefacts that arose during *de novo* genome assembly or gene prediction, but could also be due to biological reasons such as gene birth and decay (Nei and Rooney, 2005). The addition of the assembled transcripts from *N. cordifolia* and *N. noctiflora* extended 32 614 trees by at least one leaf. In total, 29 330 trees in the *N. benthamiana* phylome featured at least five species and passed all filtering criteria (see Experimental procedures): these trees were used to detect the parental progenitor and to date the hybridization event. The *N. tabacum* phylome returned a tree for 65 457 seed sequences (94.4% of genes), 47 832 of which passed all our filtering criteria.

### Phylogenetic distance between taxa

**Figure 2:**
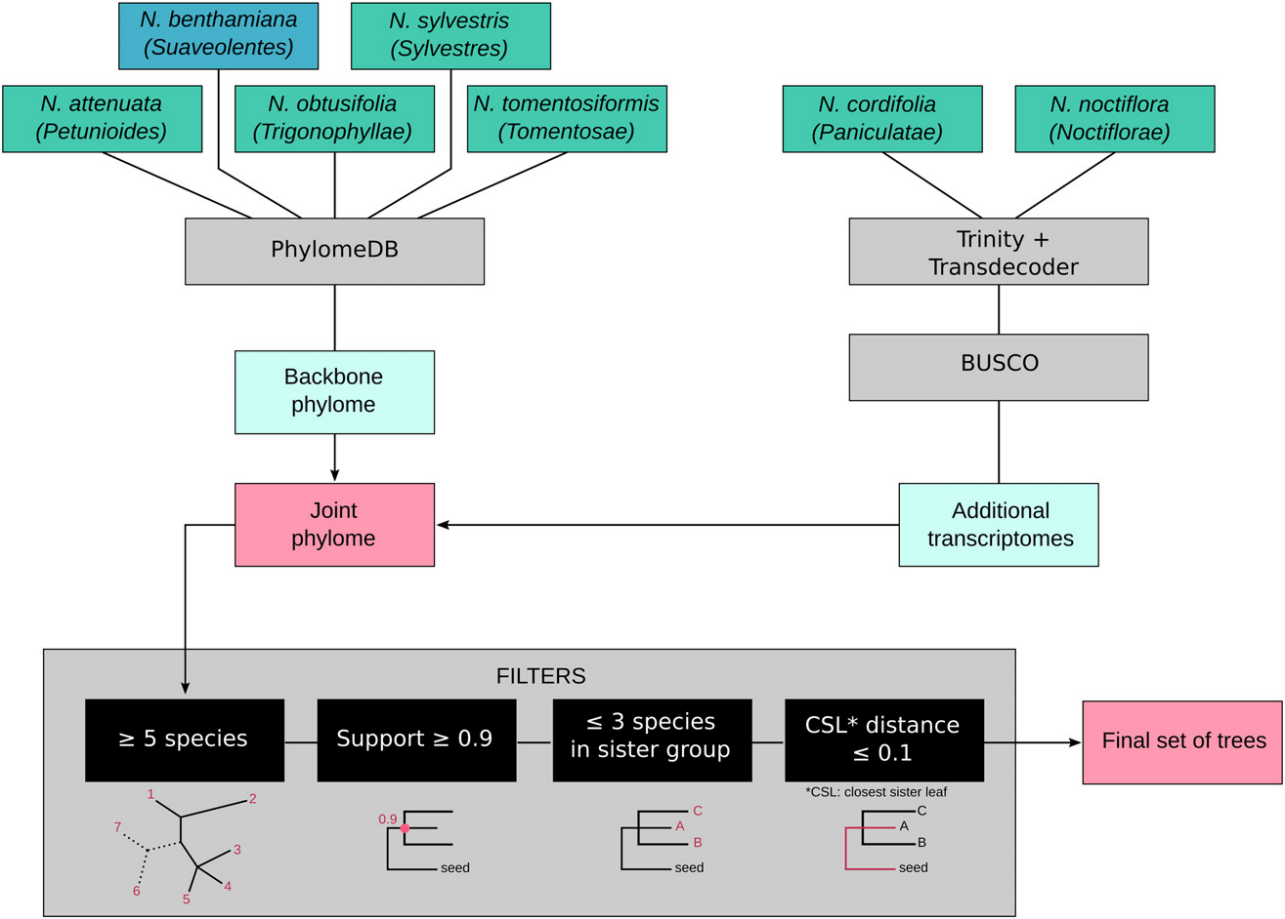
Workflow used in the generation of the *N. benthamiana* phylome. The backbone phylome was generated from gene models annotated within the sequenced genomes of five species of tobacco, including *N. benthamian*a as the seed species (blue box). To the backbone phylome, two more species (*N. cordifolia*, *N. noctiflora*) were added based on genes obtained from transcriptome assembly. Sections are indicated below the species names. Software and procedures are included in grey boxes. Red boxes indicate phylogenetic tree collections.

**Figure 3:**
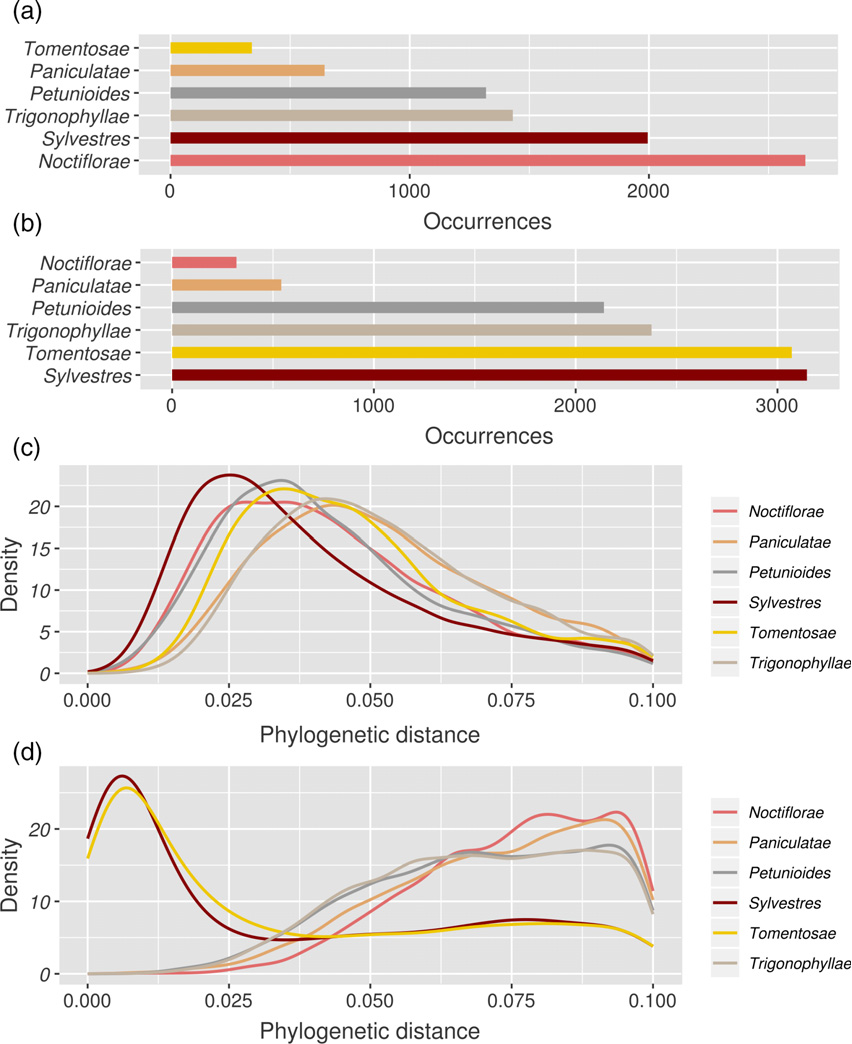
(a) Closest sister leaf (CSL) occurrences for the *N. benthamiana* phylome constructed with gene sets from different tobacco species and *N. benthamiana* genes as seed species. Colours represent different *Nicotiana* sections represented in the phylome; one species per section was included in the phylome. (b) CSLs in the *N. tabacum* phylome. (c) Distribution of phylogenetic distances expressed as substitutions per site, in a phylome based on a *N. benthamiana* seed sequence and the closest leaf for each other taxon. Colours distinguish the different *Nicotiana* sections comprised by the phylome; one species per section was included in the phylome. (d) Distribution of phylogenetic distances in the *N. tabacum* phylome.

To determine the closest relative of either *N. benthamiana* or *N. tabacum* in each gene tree, we studied the gene or gene subtree that was placed in the tree partition closest to the seed gene and belonged to a species different from the seed, using its taxonomic origin as to infer parental relationships. In 20 947 *N. benthamiana* trees and 36 239 *N. tabacum* trees these ‘closest sister leaves’ (CSLs) were not sufficiently supported, too noisy or phylogenetically too distant, and were discarded (Figure 2, see Experimental procedures). The remaining 8383 and 11 593 gene trees of *N. benthamiana* and *N. tabacum*, respectively, revealed different occurrences of each *Nicotiana* section as CSL whereby the two most recurring sections can be considered as closest relatives, that is as parental sections. In most cases the assignment to a section was straightforward as only one section was present in the closest sister subtree (5565 and 9889 gene trees in *N. benthamiana* and *N. tabacum*, respectively). In the other cases, the section of the leaf (i.e. terminal node) that showed the shortest distance to the seed gene (as inferred from the sum of the branch lengths connecting the relevant nodes) was considered. In the *N. benthamiana* gene trees, the highest occurrences as CSLs were found for sections *Noctiflorae* and *Sylvestres* (Figure 3a). In the *N. tabacum* gene trees, sections *Sylvestres* and *Tomentosae* showed the highest occurrences as CSLs, supporting previous findings (Figure 3b). This result was confirmed when analyzing the distribution of phylogenetic distances between the seed and the closest leaf from each taxon (Figure 3c). For *N. benthamiana* we identified four groups of sections, ordered by distance: (i) *Sylvestres*; (ii) *Noctiflorae* and *Petunioides;* (iii) *Tomentosae*; (iv) *Paniculatae* and *Trigonophyllae*. For *N. tabacum*, the two sections *Sylvestres* and *Tomentosae*, most likely the two parental sections as described previously, were clearly separated from all other sections (Figure 3d). Based on these data we considered sections *Noctiflorae* and *Sylvestres* as the most likely parental sections for *N. benthamiana*.

### Cluster network

**Figure 4:**
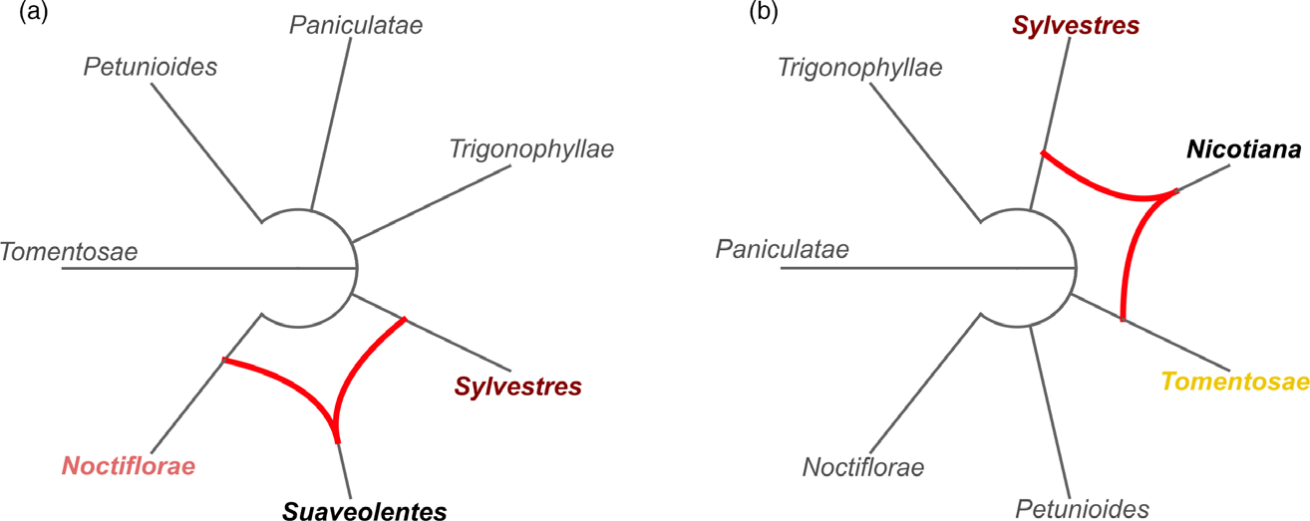
Circular cladograms showing hybridization scenarios between *Nicotiana* taxa. Branch length is not proportional to phylogenetic distance, that is only the network topology is shown. Grey lines show tree‐like branches, red lines show hybridization branches. Names of hybrid sections (*Suaveolentes* and *Nicotiana*) are indicated in black, bold characters. Names of the parental sections are indicated in bold, coloured characters. (a) Cluster network for *N. benthamiana*; (b) cluster network for *N. tabacum*.

Homoeologous genes from different subgenomes often produce phylogenetic trees that show incongruent topologies; such incongruences are a strong indicator of hybridization. Once we identified the two mostly likely parental sections for *N. benthamiana* and *N. tabacum*, we assessed whether we could observe such a hybridization with a cluster network (Huson and Rupp, 2008). We isolated phylogenetic trees whereby the CSL belonged to one of the top three sections most commonly found as CSLs, that is sections *Noctiflorae, Sylvestres* and *Trigonophyllae* for *N. benthamiana* (Figure 3a), and sections *Sylvestres, Trigonophyllae*, and *Tomentosae* for *N. tabacum* (Figure 3b)*.* Section *Trigonophyllae* was included as it was the one found most often as an alternative candidate parent throughout our analysis. From each of these two pools we created 10 random subsets of 100 trees, and we generated a cluster network from each subset. A single hybridization signal between sections *Noctiflorae* and *Sylvestres* was detected in 8/10 networks generated for *N. benthamiana*. For *N. tabacum*, a single signal was observed between sections *Sylvestres* and *Tomentosae* in 10/10 networks. Hence, we confirmed that sections *Noctiflorae* and *Sylvestres* are the most likely parental sections for *N. benthamiana*. We report the most recurrent topology for each of the two species as a circular cladogram (Figure 4).

### *N. benthamiana* subgenome analysis using transcriptome data

**Figure 5:**
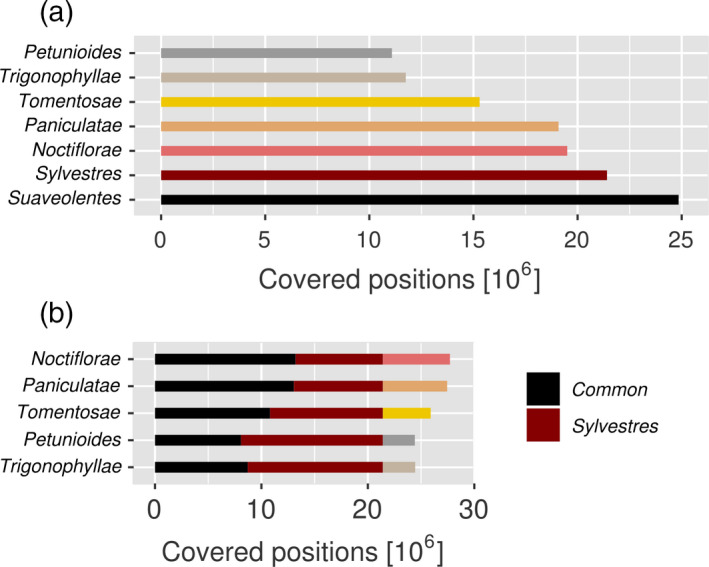
(a) Positions in annotated *N. benthamiana* exons with coverage from reads obtained by mRNA‐Seq of different *Nicotiana* species (Table 1). Colours represent different *Nicotiana* sections included in the analysis. ‘*Suaveolentes*’ refers to the *N. benthamiana* reads aligning against the *N. benthamiana* genome, that is the highest number of positions that one expects to be covered. (b) Positions covered in the parental pairs. Each line indicates a separate pair, composed of section *Sylvestres* plus another indicated section. Black colour flags positions covered by both sections, dark red indicates positions that are unique to section *Sylvestres.* The remaining part is unique to the particular indicated section (colouring as in Figure 7a).

As a different means of evaluating the origin of the *N. benthamiana* subgenomes we mapped Illumina mRNA‐Seq data from different species of tobacco against the *N. benthamiana* genome. For each diploid species of tobacco in the *N. benthamiana* phylome, we used a subset of eight million quality‐trimmed read‐pairs available from publicly available transcriptomic data (Table 1; Sierro *et al.*, 2013; Long *et al.*, 2016). After mapping, we calculated the coverage per position on 81 412 192 positions within annotated exons in the *N. benthamiana* genome based on a gene set that we had calculated previously (named ‘NibSet‐1’; Schiavinato *et al.*, 2019). Our results show that transcriptomic reads from species of the sections *Noctiflorae* and *Sylvestres* covered more positions than the other species (Figure 5a). We then determined which pair of candidate parents covered the NibSet‐1 gene set most extensively. To do so, we combined the results from the known paternal subgenome donor section *Sylvestres* with those of each other diploid species. We assessed what proportion of the NibSet‐1 exome was uniquely covered by either one of the two candidates, and by both. Again, we observed that a combination of section *Sylvestres* with section *Noctiflorae* makes the best parental pair (Figure 5b), closely followed by the pair formed with section *Trigonophyllae*; section *Petunioides* returned the lowest result.

### Subgenome intermixing

**Figure 6:**
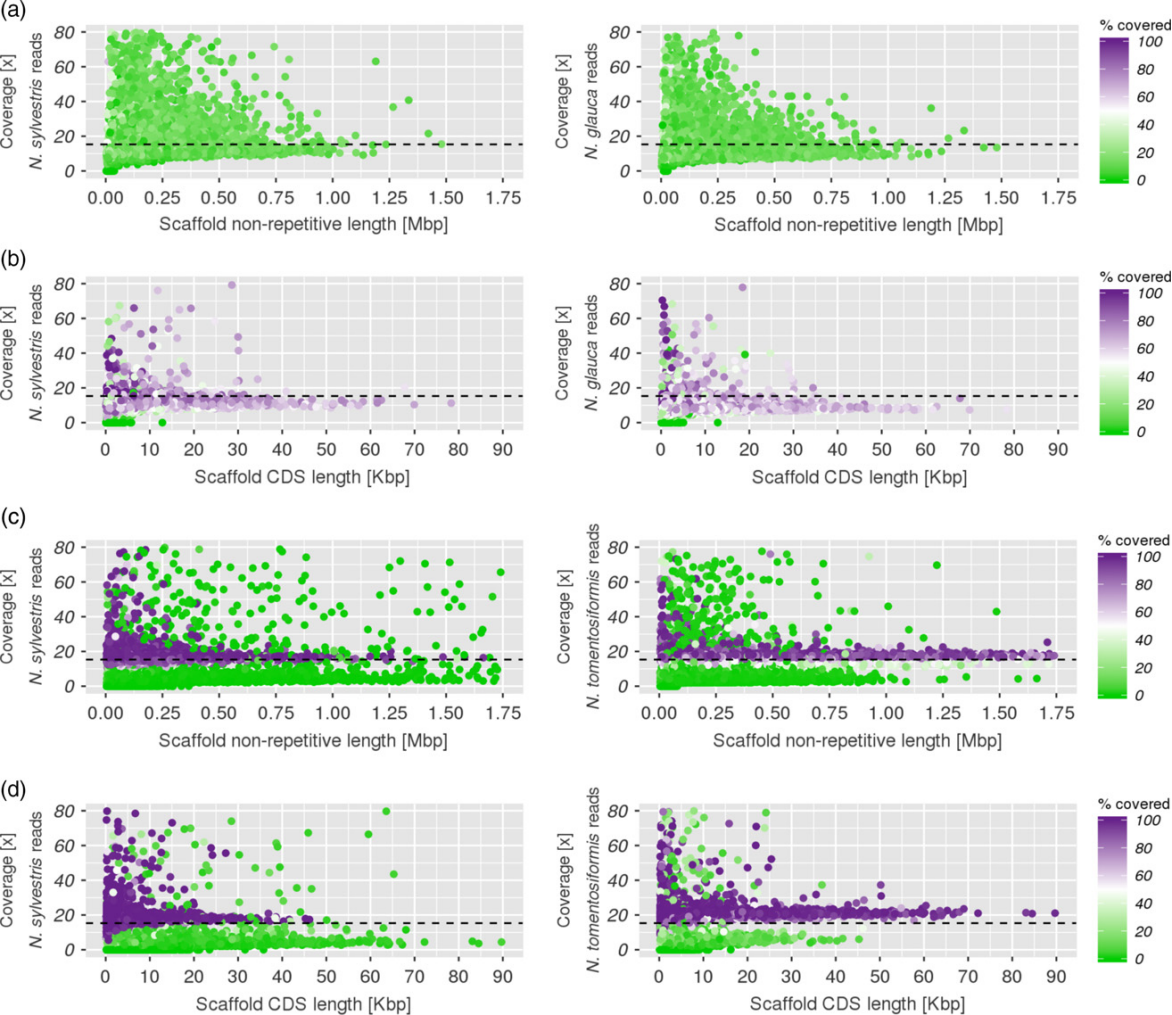
Coverage analysis in *N. benthamiana* (a, b) and *N. tabacum* (c, d) scaffolds. Each dot represents a scaffold, coloured according to the fraction of its non‐repetitive sequence that is covered by parental reads. The coverage on the y‐axis represents the scaffold's mean coverage, computed on positions with at least two mapped reads. The horizontal dashed line is the expected coverage (15.3×). Although some scaffolds showed a coverage higher than 80×, the plot has been limited to the 0‐80x range to improve readability. (a) Coverage in non‐repetitive regions of the *N. benthamiana* Nb‐1 draft genome assembly. (b) Coverage in CDS regions of the Nb‐1 assembly. (c) Coverage in non‐repetitive regions of the *N. tabacum* Nitab‐v4.5 assembly. (d) Coverage in CDS regions of the Nitab‐v4.5 assembly.

Genomic data of one of the parental sections may be used to identify the portion of the genome that originated from this parental section by mapping genomic reads onto the hybridized genome. In case the two subgenomes were still distinguishable and each assignable to one of the parents, this should be reflected in the mapping result, that is one fraction of the genome would be covered well whereas the other fraction remains sparsely covered. However, if the subgenomes were highly intermixed a clear separation would probably be impossible. To assess the level of intermixing between subgenomes we mapped genomic paired‐end reads from species affiliated with section *Noctiflorae* (*N. glauca*) and *Sylvestres* (*N. sylvestris*) onto the Nb‐1 draft genome assembly, allowing for up to 5% divergence between reads and reference. Although only <20% of the reads matched sequences of the Nb‐1 assembly, the total number of matched scaffolds comprised 95% of Nb‐1. Regardless of the species from which the reads came from, these scaffolds were only partially covered by reads (min. coverage: 1×). *N. glauca* reads covered around 12% of their length (mean: 11.9%, median: 11.0%, max: 78.0%), while *N. sylvestris* reads covered around 13% of it (mean: 13.0%, median: 11.9%, max: 78.9%; Figure 6a). When considering only coding regions (CDS), *N. glauca* reads coverage increased to 60% (mean: 60.2%, median: 63.1%, max: 100.0%), while *N. sylvestris* to 65% (mean: 65.4%, median: 69.0%, max: 100.0%; Figure 6b). In both cases, it was not possible to distinguish two separate groups of scaffolds that would represent the two subgenomes.

For comparison, we mapped genomic reads from species affiliated with sections *Sylvestres* (*N. sylvestris*) and *Tomentosae* (*N. tomentosiformis*) against the scaffolds of the *N. tabacum* Nitab‐v4.5 assembly (Edwards *et al.*, 2017), allowing a divergence of 2%, and computed the covered fraction in the same way as for *N. benthamiana*. This time, 84.0% of the *N. sylvestris* reads and 72.4% of the *N. tomentosiformis* reads were mapped, and we could clearly identify two groups of scaffolds, that is one highly covered fraction (range: 50–100%) and one poorly covered fraction (range: 0–50%; Figure 6c). Within the highly covered fraction, the *N. sylvestris* reads covered around 95% of the scaffold lengths (mean: 95.4%, median: 98.0%, max: 100.0%), while the *N. tomentosiformis* covered around 89% (mean: 88.8%, median: 93.4%, max: 99.8%). Within the poorly covered fraction, the *N. sylvestris* reads covered around 2% (mean: 1.5%, median: 0.3%, max: 49.5%), and the same for *N. tomentosiformis* reads (mean: 2.3%, median: 0.7%, max: 50.0%). Within CDS regions the distinction between the two groups was even sharper (Figure 6d). The highly covered fraction approximated a full coverage for both *N. sylvestris* (mean: 99.1%, median: 100.0%, max: 100.0%) and *N. tomentosiformis* (mean: 96.0%, median: 99.4%, max: 100.0%), while the poorly covered fraction maintained low coverage (mean: 3.4%, median: 1.6%, max: 49.7% for *N. sylvestris*; mean: 7.1%, median: 5.0%, max: 49.4% for *N. tomentosiformis*). This result confirmed, on the one hand, that mapping of genomic reads can identify separate subgenomes and suggested on the other hand that the subgenomes of *N. benthamiana* were much more intermixed than those of *N. tabacum*.

### Subgenome separation

As genomic mapping was shown to be hardly promising for the separation of highly intermixed subgenomes, we attempted to separate the two subgenomes of *N. benthamiana* using the phylogenetic information provided within its phylome. By considering only gene‐encoding regions we ruled out the high repetitive content and reduced the effect of the advanced genomic intermixing state of the two subgenomes. Out of 44 424 trees, we extracted 43 597 trees whereby each sequence in the underlying multiple sequence alignment showed, at most, 10% undetermined characters ('N'). In each tree we extracted the leaf belonging to a parental section that was closest to the *N. benthamiana* seed sequence. Such leaves were considered as reliable when they showed a node support of at least 0.9. In total, 18 071 extracted leaves belonged to section *Noctiflorae* and 15 511 to section *Sylvestres*. We refer to a seed gene as ‘orphan’ if no leaf of a parental section was contained in its tree (10 015 trees). Wherever possible we inferred the parental information for orphan trees from neighbouring genes in the genome sequence (see Experimental procedures). A maternal origin (*Noctiflorae*) was inferred for 1881 orphan genes, while a paternal origin (*Sylvestres*) was inferred for 1501 orphan genes. Our final set of assigned genes comprised 19 952 maternal genes and 17 012 paternal genes; 6633 genes remained orphans.

We used the genes' parental information to assign a parental origin to the Nb‐1 assembly scaffolds. Scaffolds containing at least one gene sum up to 95% of the total assembly size (10 363 scaffolds, mean 4.9 genes per scaffold). Scaffolds containing genes from both parents were only assigned if one parent was represented by at least 75% of the genes. Genes of unknown parental origin were ignored. In this way, we assigned a maternal origin to 1.42 Gbp (50.3%) and a paternal origin to 0.97 Gbp (34.4%) of Nb‐1 (Table 2 and Supporting Information Files S1 and S2).

**Table 2:** Parental assignment of *N. benthamiana* scaffolds

Assignment	Number of scaffolds	Total length	Fraction (%)	Avg. scaffold length	Avg. number of genes
*Noctiflorae*	3972	1 419 752 456	50.3%	357 440.2	6.2
*Sylvestres*	3259	970 554 883	34.4%	297 807.6	5.4
Orphan	3132	432 579 414	15.3%	138 116.0	2.6
Total	10 363	2 822 886 753	100.0%	272 400.5	4.9

### Dating the hybridization event

**Figure 7:**
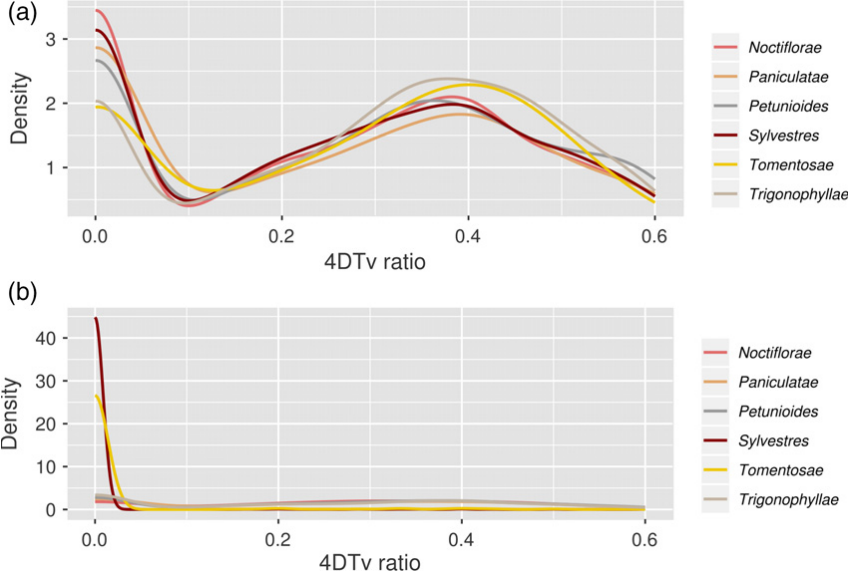
Ratio of transversions (Tv) at four‐fold degenerate (4D) sites computed between a seed sequence and its closest sister leaf (CSL) in each phylogenetic tree of phylomes comprising different species of tobacco. Colours represent *Nicotiana* sections. The ratio is obtained by dividing the number of transversions to the total number of substitutions. (a) Phylome with seed sequence from *N. benthamiana*, or (b) with seed sequence from *N. tabacum.*

To date the hybridization events that had led to the formation of *N. benthamiana* and *N. tabacum,* respectively*,* we studied four‐fold degenerate (4D) sites (Lagerkvist, 1978). 4D sites are third positions in codons that can mutate to any nucleotide without changing the translated amino acid (Topal and Fresco, 1976). Hence, they represent an ideal molecular clock in a situation in which few calibration points are available. First, we assessed the transversion (Tv) ratio at these sites, computing the 4DTv ratio in each tree with a valid CSL, that is 8383 trees in *N. benthamiana* and 11 593 trees in *N. tabacum*. A 4DTv ratio is only a relative measurement of divergence time, that is it cannot be converted into an absolute time measurement such as years. However, concordance between the relative divergence times of the parental sections could corroborate our claim on the parental section choice. Within each tree we computed the 4DTv ratio only between the seed sequence and its CSL. We applied the criteria that, firstly, an alignment needed to encompass at least five substitutions to compute a ratio, and, secondly, that seed and CSL must diverge by at most 0.05 substitutions per site. We chose such strict filtering thresholds in order to exclude genes which either evolve particularly slow or fast. We plotted the 4DTv ratios obtained from each tree to analyze their distribution. Ideally, a species 4DTv ratio is defined by a sharp peak in the distribution, meaning that most of its gene trees returned similar 4DTv ratios. Since they define relative time distances, the ideal case would be that only the two parental species have a peak at the same 4DTv ratio, confirming that they diverged at the same time from the hybrid. We were not able to distinguish *N. benthamiana*'s parents based on this criterion: the peaks obtained from most sections overlapped to a great extent, with the exception of section *Tomentosae* and *Trigonophyllae* (Figure 7a). The results obtained within the *N. tabacum* phylome instead showed a strong peak concordance only for the two parental sections (Figure 7b).

**Figure 8:**
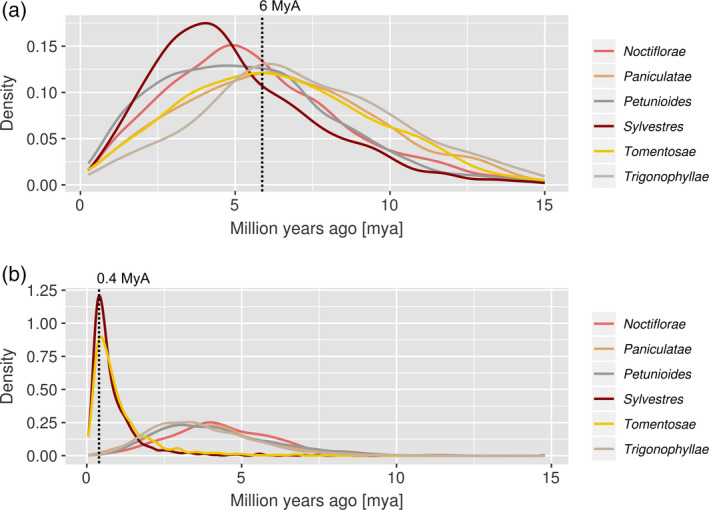
Distribution of divergence times (millions of years), obtained from the phylome trees. Colours represent *Nicotiana sections.* (a) Divergence time between *N. benthamiana* and a species of the indicated sections. (b) *N. tabacum* results. The black dashed lines indicate the latest estimates for the timing when hybridization took place in the generation of sections Noctiflorae (a) and Sylvestres (b) (Clarkson *et al*., 2017).

We then used the same sets of trees to obtain an absolute time distance between the hybrid and the parental species (i.e. a hybridization date). We used the substitutions per position at four‐fold degenerate sites (this time regardless of the nature of the substitution) as a measure of sequence divergence under neutral evolution. In each tree the value was computed only between the seed and its CSL. We divided those values by a rate of substitutions per position per generation time (5e‐09) in the range of those expected for plant nuclear genes (Wolfe *et al.*, 1989), divided by 2 to account for the parallel evolution of seed and CSL from their most recent common ancestor; the results were then scaled to millions of years and plotted to study their distribution (Figure 8). The divergence times estimated for *N. benthamiana* show that the last‐diverging sections were *Sylvestres, Petunioides*, and *Noctiflorae*; however, as for the phylogenetic distance distributions, their divergence times distributions were largely overlapping. Based on the divergence time distributions obtained from trees having either one of the two parents as CSL, we would estimate the hybridization event to have taken place 4‐5 MyA (peak: 4.9 MyA). In *N. tabacum* we observed a clear separation between the distributions of the presumable two parental sections and all other sections leading to an estimation for the hybridization event having occurred 0–0.5 MyA (peak: 0.4 MyA; Table 3).

**Table 3:** Arithmetic means of the divergence time in million years obtained from each phylome by comparing the seed sequence against the closest homologue from each taxon in the data set

Section	*N. benthamiana* (peak, MyA)	*N. tabacum* (peak, MyA)
*Noctiflorae*	4.9	4.0
*Paniculatae*	6.1	3.0
*Petunioides*	4.7	3.1
*Trigonophyllae*	6.1	3.3
*Tomentosae*	5.8	0.4
*Sylvestres*	4.0	0.4

## Discussion

### The progenitors of *N. benthamiana*

By means of a phylogenetic analysis, we have set out to identify extant species that are the closest living relatives of the parental progenitors of *N. benthamiana*. We studied a phylome constructed with gene sets from *N. benthamiana* and six diploid tobacco species. In most cases, a homologue from section *Noctiflorae* represented the CSL, followed by section *Sylvestres* (Figure 3a)*.* The strength of the hybridization signal from these candidate parental progenitors was clearly visible in the cluster network (Figure 4). Previously, the maternal parent of *N. benthamiana* has been attributed either to section *Noctiflorae* or *Petunioides*, or to one of the two having introgressed DNA from the other (Clarkson *et al.*, 2004; Kelly *et al.*, 2012). In our analysis, we provide evidence that a *Noctiflorae* species is the most probable maternal subgenome donor of *N. benthamiana*. However, we do note the presence of a plateau in the distribution of phylogenetic distances obtained from section *Noctiflorae*. The plateau encompasses the peaks corresponding to sections *Sylvestres* and *Petunioides*. We speculate that the maternal progenitor might have introgressed some DNA from an ancestor of section *Petunioides* before hybridizing with an ancestor of section *Sylvestres*, in line with previous findings (Kelly *et al.*, 2012). We noted a disproportion in the two parental CSL counts for *N. benthamiana* that could be attributed to biased fractionation of the paternally inherited subgenome (Leitch *et al.*, 2008; Petit *et al.*, 2010; Renny‐Byfield *et al.*, 2011). This is in line with our observation on subgenome intermixing, where we found that *N. sylvestris* reads cover only 10–20% of the non‐repetitive positions in Nb‐1 scaffolds. In the comparatively young allopolyploid *N. tabacum*, we did not observe such a trend: the sets of genes attributed as being derived from *Sylvestres* or *Tomentosae,* respectively*,* are similar in number (Figure 3b) and the two subgenomes can be clearly separated as inferred from mapping results (Figure 6c,d). The phylogenetic distances of *N. benthamiana* orthologues to all the other tested species were in a narrow range (Figure 3c). Even though the CSL counts showed a prevalence of section *Noctiflorae* over *Petunioides*, the close evolutionary distance between these two candidate parental species required a second line of evidence in order to strengthen our claim. When looking at the results obtained from mapping of mRNA‐Seq reads onto the Nb‐1 annotated coding regions, we could clearly choose section *Noctiflorae* over *Petunioides* (Figure 5). Taken together, our results suggested that a species from the section *Noctiflorae* was very likely to be the maternal subgenome donor in the hybridization event that resulted in section *Suaveolentes*.

### Subgenome separation

It has been shown that a 'genomic shock' (McClintock, 1984) induced by hybridization often leads to genomic rearrangements (Bashir *et al.*, 2018) paired with high retrotransposon activity (Petit *et al.*, 2010), and that, in *Nicotiana*, these dynamics correlate with hybrid age, beginning shortly after hybridization (Lim *et al.*, 2007). It is therefore expected that a hybrid as old as *N. benthamiana* would show extensive subgenomic intermixing, similar to hybrids of section *Repandae* (Lim *et al.*, 2007). Traditional methods based on read mapping were successful for subgenome separation of *N. tabacum* but failed for *N. benthamiana* (Figure 6). In young hybrids, subgenomes are more easily distinguishable due to short divergence time. We assume that intermixing of subgenomes progresses with time, and in the older hybrid *N. benthamiana* reached a level at which subgenome discrimination is far less obvious, also because both the subgenomes and the extant relatives of their parental species have been diverging for long period of time. To overcome this obstacle, we used the parental assignment of the genes in more than 40 000 trees in the *N. benthamiana* phylome, based on which we inferred parental origin for 85% of the Nb‐1 assembly. The observed disproportion in favour of maternally inherited DNA (50.3% versus 34.4%) may be a result of biased fractionation of the genome in its long‐term diploidization process (Clarkson *et al.*, 2005). We concluded that subgenome separation based on large phylogenetic data collections can overcome the difficulties encountered by read mapping methods.

### Dating of the interspecific hybridization event

For dating the *Suaveolentes* hybridization event we counted substitutions at neutrally evolving positions between orthologues in each of the trees used for counting CSL occurrences. We focused on four‐fold degenerate sites (Crick, 1968). We first looked at transversions (Tv) and transitions (Ti) at these sites. It is generally accepted that Tv are less frequent than Ti; the accumulation of transversions at four‐fold degenerate sites (4DTv) is therefore slow and can be used as a footprint of time. Moreover, it is expected that 4DTv ratios computed from extant species related to the parental progenitors are similar, as both progenitors have been separated from the hybrid for the same time. As with phylogenetic distances, our *N. benthamiana* results could not clearly separate the parental progenitors from the others (Figure 7). We note that sections *Trigonophyllae* and *Tomentosae* have a slightly larger 4DTv ratio when compared with the other four sections. Interestingly, section *Paniculatae* (represented by *N. cordifolia*) shared the same 4DTv profile with *Noctiflorae, Petunioides*, and *Sylvestres*. However, this is likely to be an artefact, as *N. cordifolia* is endemic to islands off the Chilean coast (Clarkson *et al.*, 2017). In fact, despite the similar 4DTv profile, its phylogenetic distance distribution (Figure 3c) indicates larger evolutionary distance. We speculate that sections *Noctiflorae, Petunioides*, and *Sylvestres* diverged at about the same time. For *N. tabacum,* its young age did not allow for many transversions to accumulate in the genome, therefore the peaks of the distributions regarding the parental species are close to zero.

When looking at the divergence times as computed from the *N. benthamiana* phylome, it is also complicated to distinguish between parental and non‐parental sections (Figure 8a). We clearly identified a separate peak for section *Sylvestres*, but the peaks of sections *Noctiflorae* and *Petunioides* were close. From the other analyses in our work we know that the maternal progenitor likely was a species from section *Noctiflorae.* However, its peak and the one of section *Sylvestres* do not coincide. It can be envisaged that the genomes of *N. sylvestris* and *N. noctiflora* are only modern descendants of the species that generated the original amphidiploid hybrid. Hence, their genomes might be different from their ancestral counterparts, or could have mutated at different rates. Our results place the hybridization events leading to *N. benthamiana* at 4–5 MyA and to *N. tabacum* at 0–0.5 MyA (Figure 8). The arithmetic means computed on the divergence times between each taxon and the hybrids (Table 3) were in concordance with the recent literature (Clarkson *et al.*, 2017), which suggests an age of 6 MyA for section *Suaveolentes*, with maternal and paternal sections returning different estimations (6.4 MyA and 5.5 MyA, respectively). They also show an age of 0.4 MyA for *N. tabacum,* which fits our findings. Taking these results into account, we conclude that *N. benthamiana* was likely to have originated from a hybridization event that occurred around 5 MyA.

## Experimental procedures

### Gene sets and transcriptome assemblies

We selected one *Nicotiana* species as representative for each of the eight sections (Figure 1, Table 1). We obtained gene sets for *N. attenuata* and *N. obtusifolia* (Xu *et al.*, 2017), for *N. sylvestris* and *N. tomentosiformis* (Sierro *et al.*, 2013), *N. benthamiana* (Schiavinato *et al.*, 2019), and *N. tabacum* (Edwards *et al.*, 2017) from public resources (http://nadh.ice.mpg.de/NaDH/download/; http://bioinformatics.boku.ac.at/NicBenth/Download/; https://solgenomics.net/organism/Nicotiana_tabacum/genome). For *N. cordifolia* and *N. noctiflora* we assembled gene sets from publicly available transcriptome sequencing data. Illumina mRNA‐Seq data from leaves (i.e. high‐throughput sequencing data generated on polyA + selected transcripts after conversion to cDNA) were downloaded from the NCBI sequence read archive (SRA; Leinonen *et al.*, 2011) for *N. cordifolia* and *N. noctiflora* (Table 1). We quality trimmed the reads with Trimmomatic v0.35 (Bolger *et al.*, 2014; SLIDINGWINDOW:2:20 MINLEN:50) and assembled them using Trinity v2.4.0 (Grabherr *et al.*, 2011; ‐‐no_normalize_reads ‐‐min_contig_length 200 ‐‐KMER_SIZE 25 ‐‐min_glue 2). A single transcript sequence, its CDS, and its protein translation were obtained for each gene with Trans‐decoder v3.0.1 (Haas *et al.*, 2013), using the ‐‐single_best_orf option. Sequences translating a protein shorter than 100 amino acids were removed. The protein sequences of the assembled gene sets were used for validation with BUSCO v2.0 (Simão *et al.*, 2015) and the embryophyta odb9 dataset (‐e 0.001 ‐m prot ‐f ‐‐long) downloaded from https://busco.ezlab.org/datasets/. For each of the eight gene sets, we represented each gene with its longest isoform only.

### Phylome reconstruction

We generated a comprehensive collection of phylogenetic trees (‘phylome’) for *N. benthamiana* and another one for *N. tabacum* using the phylomeDB pipeline (Huerta‐Cepas *et al.*, 2014). Based on both CDS (nt) and protein sequences (aa), the pipeline performs homology searches, multiple sequence alignments (MSA) and generates a maximum likelihood (ML) tree for each gene of the seed species (Huerta‐Cepas *et al.*, 2007; Huerta‐Cepas *et al.*, 2014). A protein MSA is generated and quality trimmed; the trimmed protein MSA is then converted to a nucleotide MSA using the nucleotide coding sequences of each gene so that silent mutations can be evaluated. We uploaded the protein sequences of *N. benthamiana* (Schiavinato *et al.*, 2019), *N. tabacum* (Edwards *et al.*, 2017)*, N. attenuata, N. obtusifolia* (Xu *et al.*, 2017)*, N. sylvestris,* and *N. tomentosiformis* (Sierro *et al.*, 2013) to the phylomeDB database, using the corresponding NCBI taxonomy IDs (Table 1, excluding *N. cordifolia* and *N. noctiflora*). Sequences yielding identical md5sums were collapsed upon upload. We specified either *N. benthamiana* or *N. tabacum* as the seed species to generate their corresponding phylomes. For *N. benthamiana* we first used the four published diploid gene sets (see above) to build a backbone phylome and then added the sequences of the two transcriptomes assembled by us, to reduce the risks of generating wrong topologies (Figure 2). Then, we generated a blast database with the protein sequences of *N. attenuata, N. benthamiana, N. obtusifolia, N. sylvestris*, and *N. tomentosiformis*, and searched in this database for homologues to the newly assembled sequences of *N. cordifolia* and *N. noctiflora* using blastp v.2.2.30 (Camacho *et al.*, 2009) with thresholds for E‐Value (1E‐03), alignment overlap (0.3) and sequence identity (30%). The coding sequence of each gene that found a homologue within these thresholds was incorporated in the corresponding gene tree using MAFFT v6.861b (Katoh and Standley, 2013). The *N. tabacum* phylome, in contrast, was calculated by including both the published and the assembled sequences at the same time.

The output of the PhylomeDB pipeline was a collection of ML trees and their underlying nucleotide MSAs. Trees and alignments of the *N. benthamiana* phylome were stored under the entry ‘phylome 817’ (http://phylomedb.org/phylome_817) in the PhylomeDB database, whereas those of the *N. tabacum* phylome were stored under the entry ‘phylome 251’ (http://phylomedb.org/phylome_251), and can be downloaded or browsed.

### Phylome analysis

**Figure 9:**
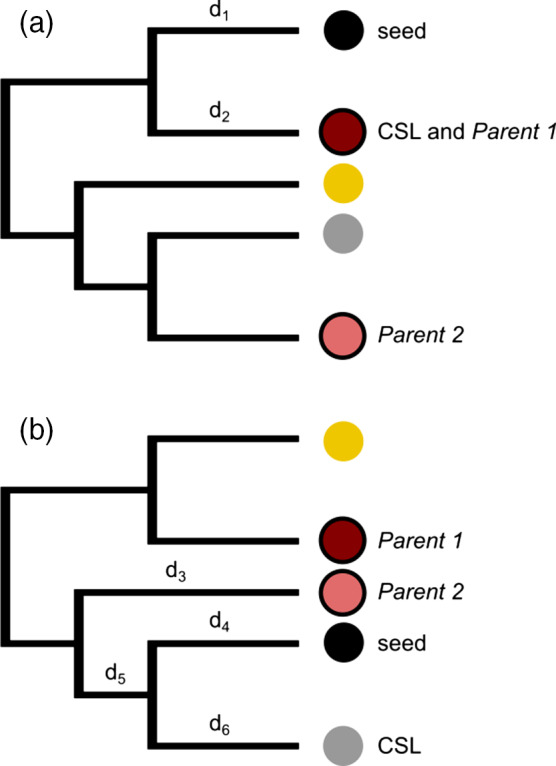
Schematic gene tree showing the seed gene, the closest sister leaf (CSL), and genes belonging to parental sections of the hybrid. Each coloured circle represents a different species. Leaves in the phylogenetic tree belonging to the candidate parents are represented in the two circles with black borders. Each *d* represents a branch length (i.e. phylogenetic distance) relevant in our analyses. (a) The CSL belongs at the same time to one of the parental sections. (b) CSL and the closest parental leaf are different from each other.

Using custom Python scripts based on ETE3 (Huerta‐Cepas *et al.*, 2016) sequences that contained >1% of undetermined bases ('N') were removed from the alignments underlying the gene trees to avoid similarity artefacts due to missing information; if the number of species in a tree fell below five in this step, we discarded the tree. From each phylome we then extracted the subset of trees that had: (i) at least five species represented, (ii) at most three species in the sister group of the seed gene, (iii) at most 0.1 phylogenetic distance (substitutions per site) between the seed gene and the CSL, and (iv) at least a support of 0.9 at the most recent common ancestor (MRCA) between the seed protein and the CSL. We extracted the CSL in each of these trees, and its phylogenetic distance (Figure 9a: CSL distance = *d*_1_ + *d*_2_; Figure 9b: *d*_4_ + *d*_6_). We computed four‐fold degenerate transversion (4DTv) ratios between seed genes and CSLs using transitions and transversions at four‐fold degenerate sites extracted from MSAs. We retained only ratios computed from at least five substitutions in trees where the seed‐CSL distance did not exceed 0.5.

To date the hybridization event we divided the total number of substitutions at four‐fold degenerate sites (regardless of the nature of the substitution) by the total number of such sites. The value was converted to million years by dividing it by a substitution rate of 5e‐09 per position per generation time, which is an accepted value for nuclear plant genes (Wolfe *et al.*, 1989).

### Cluster network

From the trees used to count CSLs we selected trees containing all the taxa and trees where the CSL belonged to one of the sections *Noctiflorae, Sylvestres*, or *Trigonophyllae* in case of the *N. benthamiana* phylome, and to one of the sections *Sylvestres, Trigonophyllae*, or *Tomentosae* in case of the *N. tabacum* phylome, respectively*.* In each tree we retained only the phylogenetically closest homologue for each taxon. From each pool of trees we extracted 100 random trees and generated a cluster network using Dendroscope 3 (Huson and Scornavacca, 2012). We iterated this procedure 10 times, generating 10 networks. We rooted the *N. benthamiana* phylome trees with section *Tomentosae*, known to be the sister taxon of the whole *Nicotiana* genus (Knapp *et al.*, 2004, p. 2004)*.* The *N. tabacum* phylome trees were rooted using section *Paniculatae*, since a species affiliated with *Tomentosae* is the paternal genome donor of the smoking tobacco. This was done merely for practical reasons, as we only generated unrooted networks. In each network we raised the cluster consensus threshold as long as at least one hybridization branch could be seen in the network. We exported each network as a circular cladogram.

### Processing and mapping of transcriptomic data

From SRA we downloaded the mRNA‐Seq datasets derived from *Nicotiana* species *N. attenuata, N. benthamiana, N. cordifolia, N. noctiflora, N. obtusifolia, N. sylvestris,* and *N. tomentosiformis*, respectively (Table 1, ‘SRA code’). Reads were quality trimmed with Trimmomatic v0.35 (ILLUMINACLIP:TruSeq2‐PE.fa:2:30:10 SLIDINGWINDOW:2:20 MINLEN:50). From each file, we extracted eight million read‐pairs and cropped them to a length of 50 nt. We mapped the reads against the *N. benthamiana* Nb‐1 draft genome assembly (Bombarely *et al.*, 2012) with HISAT2 v2.1.0 (Kim *et al.*, 2015; ‐‐score‐min L,2,‐0.4 ‐‐mp 5,5 ‐‐rdg 7,3 ‐‐rfg 7,3 ‐k 5 ‐‐no‐mixed) and kept only primary alignments (samtools view ‐F 0x0100, v. 1.3, (Li *et al.*, 2009)). We isolated covered positions within the annotated exonic Nb‐1 regions only, and extracted the coverage per position using bedtools v2.27.1 (Quinlan and Hall, 2010; bedtools genomecov ‐dz). We retained only positions with a minimum coverage of 4. We counted the base pairs that were uniquely covered by each of the tested species, or by pairs of them, using bedtools intersect.

### Subgenome intermixing

Genomic paired‐end read data were obtained from SRA for *N. glauca* (SRR6320052, SRR6320053, SRR6320054, SRR6320055, SRR6320056, SRR6320057), *N. sylvestris* (ERR274528), and *N. tomentosiformis* (ERR274540; Sierro *et al.*, 2013; Khafizova *et al.*, 2018). Reads were quality trimmed and cropped to a length of 100 nt with Trimmomatic (LEADING:15 TRAILING:15 SLIDINGWINDOW:4:20 AVGQUAL:20 CROP:100 MINLEN:50). The read length was chosen based on the sample with the shortest reads (i.e. those of *N. sylvestris* and *N. tomentosiformis*). The *N. glauca* reads were combined in a single pool. The same number of reads, that is 240 million read‐pairs, was extracted for each species. This number was chosen based on the smallest sample (*N. glauca*). The *N. glauca* and *N. sylvestris* reads were mapped against the *N. benthamiana* Nb‐1 assembly (Bombarely *et al.*, 2012), while the same *N. sylvestris* plus the *N. tomentosiformis* reads were mapped against the *N. tabacum* Nitab‐v4.5 assembly in its scaffolded version (Edwards *et al.*, 2017). Reads were mapped with HISAT2 v2.1.0 (‐‐no‐softclip ‐‐no‐spliced‐alignment ‐‐mp 6,2) using different scoring functions in Nb‐1 (‐‐score‐min L,0.0,‐0.3) and Nitab‐v4.5 (L,0.0,‐0.12), which translate into five and two mismatches per 100 positions, respectively. We retained only primary alignments for each read (samtools view ‐F 0x0100), sorted the reads by genome coordinate (samtools sort), retained reads not overlapping transposable elements (TEs; samtools view ‐L, using a bed file containing only positions outside of annotated TEs), and computed the coverage per position (samtools depth). We obtained coverage profiles over the non‐repetitive regions and over annotated CDS, respectively, referring to the published annotations for Nb‐1 (Schiavinato *et al*., 2019) and Nitab‐v4.5 (Edwards *et al*., 2017). We computed the mean coverage and the covered fraction (i.e. fraction of positions covered by reads) in each scaffold from the coverage profiles. Given the high fragmentation of the Nitab‐v4.5 assembly we excluded all scaffolds <10 kbp (24.5% of the total size of the Nitab‐v4.5 assembly, consisting of >1 million fragments).

### Subgenome separation

The parental leaves in each tree of each phylome were analyzed with a custom Python script based on ETE3 (Huerta‐Cepas *et al.*, 2016). Given the prior detection of the candidate parents in each phylome (see ‘Phylome analysis’ Experimental procedures section), the script extracted all the leaves belonging to those parental sections (i.e. *Noctiflorae* and *Sylvestres* for *N. benthamiana*, *Sylvestres*, and *Tomentosiformis* for *N. tabacum*). Those leaves were ranked by distance from the seed gene, and the one with the shortest phylogenetic distance was used to assign a parental origin to the gene. We note that the closest parental leaf can either be the CSL (Figure 9a, distance: *d*_1_ + *d*_2_) or different from the CSL (Figure 9b, distance: *d*_3_ + *d*_4_ + *d*_5_). Genes without a parental assignment were termed ‘orphan genes’, and the parental origin was inferred from the parental assignment (if any) of the upstream and downstream gene in the genome. If the neighbouring genes had the same parental assignment based on their phylome trees the assignment was transferred to the orphan gene; otherwise the gene was left as ‘orphan’. If only either one of the upstream or downstream genes was assigned to a parental section the script transferred the parental origin of this assignment. All the assigned genes within a scaffold were used to assign the scaffold to a parental origin: if at least 75% of its parentally‐assigned genes showed the same origin the scaffold was assigned to that parent.

### Software and hardware specifications

The code for this study was written in a series of Bash scripts, Python v2.7 scripts, and SoS Notebooks (Peng *et al.*, 2018); the Notebooks featured kernels for Python v3.6, R v3.4.0, and the Bash shell. All plots were generated using ggplot2 (Wickham, 2016). The phylomes were generated on an HPC cluster, using a node consisting of 64 cores (2.70 GHz) and 500 GB of RAM. The generation of one phylome took approximately 2 weeks of continuous computation using all the available cores. The phylome analyses were performed on one node featuring 24 cores (2.40 GHz) and 500 GB of RAM.

## Author contributions

TG, JCD, and HH designed experiments and supervised the study, MS analyzed and interpreted the data, MMH contributed to phylome analyses, MS, HH, and JCD wrote the manuscript with contributions from TG and MMH, all authors approved the final version of the manuscript.

## Conflicts of interest

None declared.

## Supporting information


**File S1.** Assigned parental origin of *N. benthamiana* genes.

**File S2.** Assigned parental origin of *N. benthamiana* Nb‐1 scaffolds.

## Data Availability

Trees and alignments for each gene of the *N. benthamiana* phylome are stored under the entry ‘phylome 817’ in the PhylomeDB database (http://phylomedb.org/). Trees and alignments for each gene of the *N. tabacum* phylome are stored under the entry ‘phylome 251’. Maternal, paternal, and orphan genes are listed in Supporting Information File S1. Parental assignments for scaffolds containing genes are listed in File S2. NibSet‐1 gene models can be downloaded from our data repository (http://bioinformatics.boku.ac.at/NicBenth/Download/).
