# Supplementary material for: Parental origin of the allotetraploid tobacco Nicotiana benthamiana
Source: Plant J. 2020 Jan 13;102(3):541–54. doi: 10.1111/tpj.14648 (PMC7317763; doi:10.1111/tpj.14648)
Supplement: Supplementary file 3 [file TPJ-102-541-s003.docx]

**Additional File 1**. Assigned parental origin for *N. benthamiana* genes contained in the NibSet-1 annotation (Schiavinato *et al.*, 2019) performed on the Nb-1 draft genome assembly (Bombarely *et al.*, 2012).

**Additional File 2**. Assigned parental origin for *N. benthamiana* Nb-1 scaffolds of the Nb-1 draft genome assembly (Bombarely *et al.*, 2012).
